# Surgical outcomes and indications for saccular abdominal aortic aneurysm repair: a systematic review

**DOI:** 10.1590/1677-5449.202401642

**Published:** 2025-05-30

**Authors:** João Alfredo Schiewe, Victoria Lebedenco Barbosa, João Eduardo Herrero Lima, André Brusamolin Moro, Victor Johanes Seidel, Livia Hoyer Garcia Miranda, Francisco José Fernandes Alves, Jeferson Freitas Toregeani

**Affiliations:** 1 Universidade Estadual do Centro-Oeste, Guarapuava, PR, Brasil.; 2 Centro Universitário da Fundação Assis Gurgacz, Cascavel, PR, Brasil.; 3 Hospital Estadual Vila Alpina, São Paulo, SP, Brasil.; 4 Faculdade de Medicina de Marília, Marília, SP, Brasil.; 5 Serviço de Cirurgia Vascular Elias Abrão, Curitiba, PR, Brasil.; 6 Universidade Estadual do Oeste do Paraná, Cascavel, PR, Brasil.

**Keywords:** abdominal aortic aneurysm, saccular aneurysm, postoperative complications, endovascular aneurysm repair, risk assessment, aneurisma da aorta abdominal, aneurisma sacular, complicações pós-operatórias, correção endovascular de aneurisma, medição de risco

## Abstract

Indications for surgical repair of saccular abdominal aortic aneurysms lack satisfactory evidence, and the risk of rupture has been questioned. We conducted a systematic review assessing surgical outcomes following repair of this condition. Eight studies were included, totaling 540 patients. Endovascular repair was the most common approach. Complications occurred in 18.99% of the patients, and unfavorable surgical outcomes occurred in 3.15%, of which cardiac and pulmonary comorbidities were the most frequent. Aneurysms with smaller diameters presented fewer complications and unfavorable surgical outcomes than those with larger diameters. The ideal threshold for repair remains uncertain. Although diameter is an important indicator, other factors should be considered. These aneurysms should be treated electively at earlier stages due to their uncertain rupture risk and the higher prevalence of complications at larger diameters. Further research is needed to establish clear treatment guidelines for this condition.

## Resumo

As indicações cirúrgicas para o reparo de aneurismas saculares da aorta abdominal ainda carecem de evidências satisfatórias, e o risco de ruptura dessas lesões tem sido questionado. Esta revisão sistemática avaliou os resultados cirúrgicos após o reparo dessa condição. Foram incluídos oito estudos, totalizando 540 pacientes, sendo o reparo endovascular a técnica mais utilizada. Complicações ocorreram em 18,99% dos casos, enquanto a taxa de desfechos cirúrgicos desfavoráveis foi de 3,15%. As comorbidades cardíacas e pulmonares foram as mais prevalentes entre os pacientes. Aneurismas com diâmetros menores apresentaram menos complicações e resultados cirúrgicos desfavoráveis ​​em comparação aos de maior diâmetro. O limiar ideal para a indicação cirúrgica ainda é incerto. Embora o diâmetro seja um indicador importante, outros fatores devem ser considerados na decisão terapêutica. Aneurismas saculares devem ser tratados eletivamente em estágios mais precoces, devido ao risco incerto de ruptura e à maior incidência de complicações quando operados em diâmetros avançados. Novas pesquisas são necessárias para estabelecer diretrizes claras para o tratamento dessa condição.

## INTRODUCTION

Arterial aneurysms are characterized by focal dilations with a diameter > 50% of the adjacent blood vessel diameter.^1^ In the aorta, the abdominal region is the most commonly affected by true aneurysms, with diameters ≥ 3 cm considered to be aneurysms in most patients.^2^ Abdominal aortic aneurysms (AAAs) can be classified as suprarenal, juxtarenal, pararenal, or infrarenal, with the latter having a higher prevalence, possibly due to its distinct embryological origin.^1-3^

The morphology of AAAs is a critical factor in clinical evaluation and treatment planning, since they can be either fusiform or saccular.^4^ Fusiform aneurysms are characterized by uniform and symmetrical dilation of the aortic wall, resulting in a cylindrical or tubular configuration.^4^ Saccular aneurysms present with localized and asymmetrical dilation, forming a sac-like projection.^4^ Most AAAs are the fusiform type, only approximately 5% are the saccular type.^4^

Clinical findings consistent with the condition date back to the times of Hippocrates (460 BC-377 BC), who described an unusual abdominal pulsation, a finding later elaborated on by Giovanni Morgagni.^5^ However, the first documented description of an AAA comes from Andreas Vesalius, the famous Renaissance anatomist.^6^ Various attempts at AAA repair have been made, with the first successful abdominal aortic ligature performed by Rudolph Matas in April 1923.^6^ Since then, surgical techniques for AAA repair have evolved, with current options including both open and endovascular surgery, the latter emerging as an established, less invasive technique involving significantly improved efficacy and safety indicators compared to traditional open surgical approaches.^7^

Risk factors for AAAs have been reported since 1958 and are now well elucidated.^8^ Important risk factors include smoking, advanced age, male sex, poor diet, sedentary lifestyle, dyslipidemia, hypertension, and genetic predisposition.^9-11^ The pathophysiology of AAA is associated with inflammatory processes predominantly involving the Th2 system, leading to apoptosis of smooth muscle cells in the vascular wall and degradation of the extracellular matrix.^12,13^ These events culminate in tissue remodeling and weakening of the arterial wall.^13^ Atherosclerosis may also influence this process, since atherosclerotic plaques can cause the release of metalloproteinases through shear stress.^12^ Moreover, the expansion of the intima layer as a result of atherosclerosis, causing hypoxia and cell death, can lead to focal thinning of the artery, further explaining saccular AAA (SaAAA).^12^

Primary indications for surgical repair of AAA include large fusiform aneurysms or those with rapid growth, the presence of complications and/or symptoms, and saccular aneurysms.^14,15^ These considerations are based on the main complication associated with aneurysms, namely rupture.^16^ The most validated predictive factor for this complication is the baseline diameter of the aneurysm.^16^ Factors such as female sex, rapid growth, smoking, hypertension, and cardiac, renal, and pulmonary comorbidities also increase the risk of rupture.^16-18^ Furthermore, surgery-related complications, as well as mortality, play a crucial role in indicating AAA surgery.^19^ Complications such as bleeding, infections, and, in endovascular repair, endoleaks and endograft occlusions, can increase the morbidity and mortality associated with surgery.^19^ Additionally, direct operative mortality and long-term mortality can be determining factors in assessing the risks and benefits of surgical repair.^19^

However, indications for the surgical repair of SaAAA are unclear and lack satisfactory evidence, especially regarding the diameter at which surgical intervention should be performed.^14,16^ SaAAAs are often indicated for surgical repair regardless of their size, based on the justification that they become symptomatic and are more prone to rupture at smaller diameters than the fusiform type due to greater wall stress.^16,20,21^ However, studies have raised doubts about their propensity to rupture, suggesting that saccular form alone does not necessarily increase this risk.^22,23^

The optimal management strategy for SaAAAs thus remains the subject of ongoing investigation and debate.^14,16^ It is fundamental to weigh the risk of complications and mortality in this pathology to determine the ideal timing of surgical repair, considering both the risk of aneurysm rupture if left untreated and the risk of surgery-related complications.^19^

For these reasons, the present review investigated outcomes related to elective surgical repairs specifically of SaAAAs, primarily to define an acceptable threshold for repair, as well as to fill in evidence gaps on this topic.

## METHODS

This systematic review on the surgical treatment of SaAAAs was conducted in accordance with the Cochrane Collaboration^24^ and the Preferred Reporting Items for Systematic Reviews and Meta-Analysis guidelines.^25^ A predefined protocol was established and prospectively registered in the Prospective Register of Systematic Reviews (registration number CRD42024513613).

### Database and search strategy

The Embase, PubMed (MEDLINE), and Cochrane Library electronic databases were searched. Keywords were combined with the Boolean operators AND and OR as follows: *"endovascular aneurysmal repair" OR "open aneurysmal repair" OR "open surgery" AND "abdominal aorta" OR "aortic aneurysm" OR "aortic rupture" OR "abdominal aortic aneurysm" AND "saccular".* The references of included articles were also searched.

### Search strategy and data collection

The inclusion criteria were: (1) randomized clinical trials or cohort studies; (2) studies involving endovascular repair of SaAAAs; (3) studies involving open surgery for repair of SaAAAs; and (4) studies reporting clinical outcomes of interest. The exclusion criteria were: (1) studies with overlapping surgical indications; (2) studies with surgical indications unrelated to saccular morphology; (3) studies that reported repair outcomes exclusively in ruptured aneurysms; and (4) studies that did not meet the intervention and population criteria of interest. Studies with overlapping surgical indications were excluded to prevent variability in the results and ensure the clinical relevance of findings specific to SaAAAs. The primary outcome was perioperative and postoperative complications. Secondary outcomes were: (1) mean maximum diameter of the aneurysm; (2) unfavorable surgical outcome; (3) surgery time; (4) hospital stay; (5) follow-up time; and (6) aorta-related deaths.

Two authors (JAS and JEHL) independently selected the studies. The data were then extracted and recorded by the same authors, which were then reviewed by a third investigator (VLB). The variables of interest included patient and procedure characteristics and follow-up data. A meta-analysis was not planned due to the expected lack of randomized clinical trials.^14^

### Quality assessment and risk of bias

We used the Cochrane Collaboration’s ROBINS-I tool^26^ to assess the risk of bias in observational studies and individual study quality. Conflicts were resolved through discussion and the involvement of a fourth author.

### Statistical analysis and data synthesis

The characteristics and outcome data of the included studies were collected, grouped, and compared. Continuous variables are presented as mean (SD). Categorical variables are presented as number (%). Fisher’s exact test was performed in RStudio 2023.03.0. *P*-values < 0.05 indicated statistical significance. The odds ratio was calculated from the combined data of the studies included in the analysis, presenting a 95% CI.

## RESULTS

### Research results and description of the selected studies

**Figure 1:**
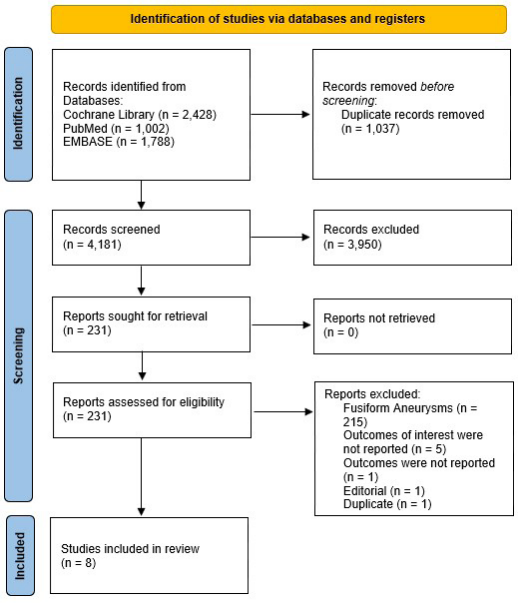
Preferred Reporting Items for Systematic Reviews and Meta-Analysis flow diagram of study screening and selection.

The initial search resulted in 5,218 studies. After removing duplicates and unrelated publications, 231 studies, which were reviewed regarding the inclusion criteria. Of these, 8 underwent qualitative and quantitative analysis (Figure 1). Table 1 summarizes the risk of bias assessment using the ROBINS-I tool. While individual domains varied between studies – ranging from low to critical in specific categories – the overall risk of bias for all studies was consistently rated as moderate. Further details of domain-specific assessments are provided in Table 1.

**Table 1:** Risk of bias assessment across domains and overall ratings using ROBINS-I.

Study	Bias due to confounding	Bias in selection of participants into the study	Bias in classification of interventions	Bias due to deviations from intended interventions	Bias due to missing data	Bias in measurement of outcomes	Bias in selection of the reported result	Overall Bias
Jones et al. (2014)^27^	Moderate	Moderate	Moderate	Low	Moderate	Low	Moderate	Moderate
York et al. (2002)^28^	Critical	Low	Low	Low	Moderate	Moderate	Moderate	Moderate
Lomazzi et al. (2022)^29^	Low	Low	Low	Low	Moderate	Low	Moderate	Moderate
Engelberger et al. (2020)^30^	Low	Low	Low	Low	Moderate	Moderate	Moderate	Moderate
D'oria et al. (2019)^31^	Moderate	Low	Low	Low	Moderate	Low	Moderate	Moderate
Mizoguchi et al. (2021)^32^	Low	Low	Moderate	Low	Moderate	Moderate	Moderate	Moderate
Karthaus et al. (2019)^20^	Low	Low	Low	Low	Low	Low	Moderate	Moderate
Blum et al. (1996)^33^	Moderate	Low	Moderate	Moderate	Moderate	^Moderate^	Moderate	Moderate

### General characteristics of the selected studies

A summary of the selected studies is shown in Table 2. Given the variation in the number of patients in each study, the data are presented in both absolute numbers and percentages to prevent misinterpretation of extreme results. The total sample size was 540 patients who underwent surgical repair of SaAAA. The pooled mean age was 74.05 (SD, 7.83) years. Patient sex was reported for 522 patients in 5 studies, of whom 432 were men (82.76%). Only one^20^ included study reported patients undergoing open surgery (12.78%); the patients in the others underwent endovascular repair (87.22%). The pooled maximum mean diameter was 49.07 (SD, 11.22) mm. One study^33^ included both saccular and fusiform aneurysms, with the mean age and diameter values being representative of the total population; however, the reported outcomes were exclusively for the 3 cases of saccular aneurysms. The mean hospitalization time and follow-up time varied significantly. The mean hospitalization time ranged from 1.6 days to 13 days, while the mean follow-up time ranged from 1 month to 41.25 months.

**Table 2:** Characteristics and outcomes of included studies.

Author and year	N° of Patients	Mean Age (years)	Sex (M/F)	Mean Maximum Diameter (mm)	Unfavorable Surgical Outcome	Surgery Time (minutes)	Hospital stay (days)	Follow-up Time (months)	Complications	Aorta-related Deaths
Jones et al. (2014)^27^	6	71.75±10.4	5/1	37.25±9.8	0 (0.0%)	127.5±67.8	2.25±1.2	3.33±3.8	0 (0.0%)	0
York et al. (2002)^28^	5	61.6±5.64	4/1	37±2	0 (0.0%)	96±41	1.6^b^	6.1±4.43	1 (20.0%)	0
Lomazzi et al. (2022)^29^	120^a^	75±8.2	103/17	38±10.5	0 (0.0%)	137±75	5.3±3.7	27.8±41.6	21 (17.5%)	0^g^
Engelberger et al. (2020)^30^	9	74.5±8	*N.S.*	41.1±14.5	1 (11.1%)	*N.S.*	*N.S.*	41.25±23.7	2 (22.2%)	0
D'oria et al. (2019)^31^	3	78.5±0.6	*N.S.*	45.5±4.3	0 (0.0%)	<70	2^c^	15±3.9	0 (0.0%)	0
Mizoguchi et al. (2021)^32^	6	71.25 ±8.2	*N.S.*	48.75±10.9	0 (0.0%)	176±59	13±6.5	22.5±11.5	2 (33.3%)	0
Karthaus et al. (2019)^20^	388	74±7.4	318/70	53±11.4	16 (4.1%)	*N.S.*	4.3± 7.42	1^d^	76 (19.6%)	-
Blum et al. (1996)^33^	3	68±30.95	*N.S.*	55±19.81	0 (0.0%)	35-150^a^	6.5±1.8	4.45±2	*N.S.*	0

Continuous variables are presented as mean ± standard deviation. Categorical variables are presented as number (percentage). ^a^ Only the maximum and minimum times were reported; the median was not provided; ^b^ Only the mean time was provided; ^c^ All patients were hospitalized for this period; ^d^ All patients were assessed 1 month after surgery. *N.S.*: not specified.

### Surgical outcomes

Four studies^27-29,32^ calculated the mean (SD) surgery time, which ranged from 98.7 to 176 minutes. Two studies^20,30^ did not report the mean surgery time. D'oria et al*.*^31^ only indicated that all surgeries were performed in < 70 minutes, while Blum et al*.*^33^ reported a range of 35 to 150 minutes.

Only two studies^20,30^ reported unfavorable surgical outcomes, totaling 17 cases (3.15%). Engelberger et al*.*^30^ reported a single reintervention, and Karthaus et al*.*^20^ reported 15 reinterventions and 1 conversion to open surgery.

Complications were mentioned in 7 studies,^20,27-32^ with 5 reporting their occurrence (Table 3).^20,28-30,32^ A total of 102 complications were reported (18.99%) in this review. Of these, 65 were classified as unspecified in the original study.^20^ Eleven patients required blood transfusions. Twelve endograft occlusions and 7 endoleaks were reported. The following were also reported: 2 cases of acute renal failure, 2 urinary tract infections, 1 endograft infection, 1 resuscitation, and 1 groin hematoma. No procedure-related deaths were reported. We did not consider the deaths reported in 1 study because we were unable to distinguish between those related and unrelated to aortic pathology.^20^ Of the 120 patients reported by Lomazzi et al*.,*^29^ 10 presented with ruptured aneurysms upon admission. The complications mentioned in the study were not distinguished according to ruptured and non-ruptured aneurysms. However, we chose to include them, considering that the majority of patients (91.67%) did not have a rupture. Therefore, it is possible that the complications reported in this study are overestimated.

**Table 3:** Recorded perioperative and postoperative complications.

	**York et al.** **^28^** **(n=5)**	**Lomazzi et al.** **^29^** **(n=120)**	**Engelberger et al.** **^30^** **(n=9)**	**Mizoguchi et al.** **^32^** **(n=6)**	**Karthaus et al.** **^20^** **(n=388)**
Complications					
Endoleak	1 (20%)	-	2 (22.22%)	-	-
Endograft occlusion		4 (3.33%)	-	2 (33.33%)	-
Blood transfusion	-	11 (9.17%)	-	-	-
Endograft infection	-	1 (0.83%)	-	-	-
Groin hematoma	-	1 (0.83%)	-	-	-
Acute renal failure	-	2 (1.67%)	-	-	-
Urinary tract infection	-	2 (1.67%)	-	-	-
Unspecified	-	-	-	-	65 (16.75%)

Data are presented as number (percentage).

**Figure 2:**
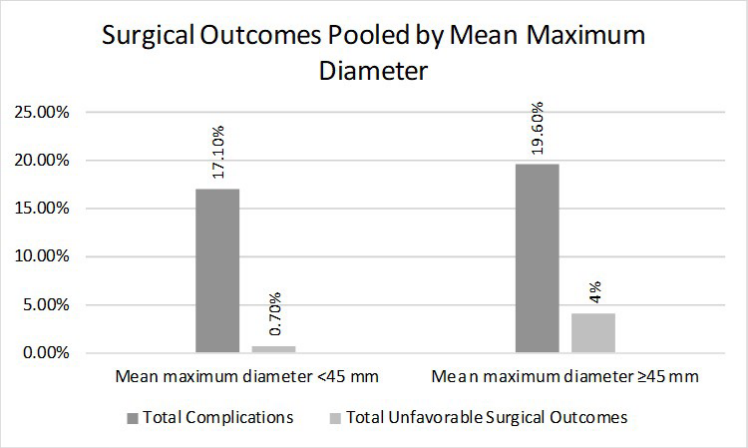
Surgical outcomes pooled by mean maximum diameter.

When the studies were categorized based on the mean maximum diameter, (< or ≥ 45 mm), 24 complications (17.1%) occurred in the group with the smaller diameter and 78 complications (19.6%) occurred in the group with the larger diameter. Regarding unfavorable surgical outcomes, only 1 case (0.7%) was observed in the smaller diameter group, while 16 cases (4%) occurred in the larger diameter group (Table 4). The smaller diameter group had a lower incidence of complications and unfavorable surgical outcomes than the larger diameter group (Figure 2).

**Table 4:** Comparison of surgical outcomes based on Aneurysm Diameter Categories.

	Mean maximum diameter <45 mm	Mean maximum diameter ≥45 mm	Odds Ratio (95% CI)	p-value
**Complications**	11 Blood transfusions	65 Unspecified complications	1.18 (0.71 – 1.96)	0.62
	4 Endograft occlusions	8 Endograft occlusion		
	3 Endoleaks	4 Endoleaks		
	2 Acute renal failures	1 resuscitation		
	2 Urinary tract infections	(n = 397)		
	1 Groin hematoma			
	1 Endograft infection			
	(n = 140)			
**Unfavorable Surgical Outcomes**	1 Reintervention	15 Reinterventions	5.78 (0.76 – 44.08)	0.09
	(n = 140)	1 Conversion to open surgery		
		(n = 400)		

The statistical analysis was made using Fisher's exact test in RStudio 2023.03.0.

**Figure 3:**
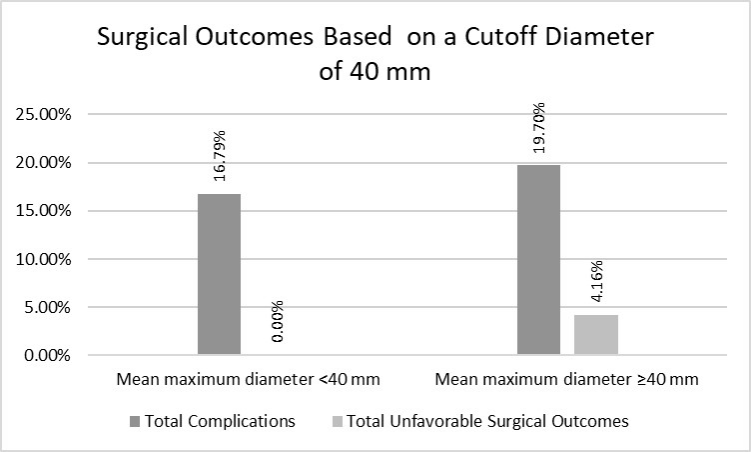
Surgical outcomes based on a cutoff diameter of 40 mm.

We also divided the maximum mean diameters into groups of < and ≥ 40 mm. In the < 40 mm group, 22 complications (16.79%) occurred, while in the ≥ 40 mm group, 80 complications (19.70%) occurred. No unfavorable surgical outcomes were observed in the smaller diameter group, whereas 17 (4.16%) occurred in larger diameter group (Figure 3).

Discussion of unfavorable outcomes is based on data from 2 studies,^20,30^ since these were the only ones to report such outcomes. The other studies in our analysis did not report unfavorable outcomes (Table 2).

### Associated comorbidities

Four studies^20,27-29^ addressed comorbidities associated with SaAAAs, totaling 519 patients, as shown in Table 5. All studies identified cardiac comorbidities, especially hypertension, coronary artery disease, and atrial fibrillation. Hypertension, specifically, was the most commonly reported, occurring in 110 patients (21.19%). Karthaus et al*.*^20^ did not describe cardiac comorbidities in their patients.

**Table 5:** Patients with comorbidities associated with saccular abdominal aortic aneurysms.

	Jones et al.^27^ (n = 6)	York et al.^28^ n = 5)	Lomazzi et al.^29^ (n = 120)	Karthaus et al.^20^ (n = 388)
Comorbidities, n (%)				
Hypertension	4 (66.67%)	3 (60%)	3 (2.5%)	-
Renal dysfunction	1 (16.67%)	1 (20%)	21 (17.5%)	-
Dyslipidemia	3 (50%)	-	55 (45.83%)	-
Obesity	1 (16.67%)	-	11 (9.17%)	-
Coronary artery disease	-	2 (40%)	54 (45%)	-
COPD	-	3 (60%)	35 (29.17%)	-
Diabetes	-	1 (20%)	7 (5.83%)	-
Peripheral artery disease	1 (16.67%)	-	-	-
Atrial fibrillation	-	-	18 (15%)	-
Unspecified cardiac comorbidity	-	-	-	238 (61.34%)
Unspecified pulmonary comorbidity	-	-	-	116 (29.90%)

Data are presented as number (percentage). COPD: chronic obstructive pulmonary disease.

Additionally, pulmonary comorbidities were frequently observed, occurring in 154 patients (29.67%), including 38 (7.32%) specifically diagnosed with chronic obstructive pulmonary disease. Other types were not specifically mentioned. Dyslipidemia and obesity were reported in 2 studies,^27,29^ corresponding to 58 (11.17%) and 12 (2.31%) patients, respectively.

Furthermore, renal dysfunction prior to the repair was reported in all studies except Karthaus et al*.,*^20^ totaling 23 patients (4.43%). Diabetes was identified in 2 studies, totaling only 8 patients (1.54%). Finally, a study^27^ reported 1 patient (0.19%) with peripheral arterial disease as a comorbidity associated with SaAAA.

## DISCUSSION

Cardiac and pulmonary comorbidities were the most commonly reported types in this review, suggesting that they are risk factors for the condition. No study reported deaths related to aortic pathologies during follow-up. Moreover, when grouped by mean diameter, complications and unfavorable surgical outcomes were more prevalent when larger-diameter SaAAAs were surgically repaired.

The comorbidities identified in the selected studies, along with advanced age and male sex, are in line with the risk factors traditionally associated with AAA.^10,14,34,35^ The high prevalence of hypertension, coronary artery disease, dyslipidemia, and obesity among the patients reinforces the role of these comorbidities as etiological factors in the development of both fusiform and SaAAA.^34,35^ Additionally, according to the European Society for Vascular Surgery, coronary artery disease has emerged as the leading cause of mortality in patients undergoing AAA repair.^35^ Renal disease, diabetes mellitus, and chronic obstructive pulmonary disease also appear to be associated with increased mortality rates after surgical intervention.^35,36^

Chronic obstructive pulmonary disease seems to be correlated with high rates of aneurysm growth and increased risk of rupture at smaller diameters.^35^ Although diabetes mellitus was cited as an associated comorbidity in 2 studies, it has been classified as a negative risk factor for AAA.^10^ Furthermore, peripheral arterial disease seems to have a low prevalence in individuals with a genetic predisposition to aortic aneurysms,^37^ being the least reported comorbidity in this review.

A total of 87.22% of the patients in this study underwent endovascular repair. In studies addressing this technique alone, only 0.67% of the patients had unfavorable surgical outcomes, highlighting the effectiveness of endovascular repair for SaAAA. This type of repair has undergone significant development since its introduction, with recent results showing technical improvements and favorable mortality indicators.^38^ It was also noted that no aorta-related deaths were recorded, suggesting a positive prognosis for patients undergoing endovascular repair. However, despite these favorable results, it is worth noting that the endovascular technique is not free from risks and complications.^39^ Additionally, the absence of mortality recorded in this review, both in the perioperative period and during follow-up, does not represent the real risk of mortality from this procedure. This result may have been due to the limited follow-up period and small population sample in some studies.^20,27,28,30-33^

Karthaus et al*.,*^20^ a large cohort study, proposed 45 mm as an acceptable threshold for surgical SaAAA repair. Ozawa et al*.*^36^ found comparable sensitivity between a threshold of 55 mm for fusiform aneurysms - accepted as a threshold for surgical repair of fusiform AAA in men^14^ - and 43 mm for SaAAA when examining the risk of rupture between AAA morphologies. Based on this, we chose to group unfavorable surgical outcomes and complications into diameters < or ≥ 45. In this context, studies in which the SaAAAs had a smaller mean diameter (<45 mm) showed superior surgical outcomes and fewer complications than those with a larger mean diameter (≥45 mm), even though Lomazzi et al*.*^29^ may have overestimated complications in the smaller diameter group (Figure 2). The odds ratio analyses (95% CI) revealed values of 1.18 (0.71 – 1.96) with *p* = 0.62 and 5.78 (0.76 – 44.08) with *p* = 0.09 for the 2 variables (Table 4). While these findings lack statistical significance, they still contribute to a broader understanding of the context.

By further reducing the cutoff value of the mean maximum diameter from 45 mm to 40 mm, we observed that the pattern of unfavorable surgical outcomes and complications remained consistent with the previously observed results. The group with a smaller mean maximum diameter had fewer unfavorable surgical outcomes and complications (Figure 3). At this threshold diameter, the disparity in unfavorable surgical outcomes between two groups became statistically significant (*p* = 0.01), with OR = 11.73 (0.70 – 196.34), indicating that SaAAA repairs conducted at early stages had better surgical outcomes (Table 6).

**Table 6:** Unfavorable surgical outcomes and complications based on a cutoff diameter of 40 mm.

	Mean maximum diameter <40 mm	Mean maximum diameter ≥40 mm	Odds Ratio (95% CI)	p-value
**Complications**	11 Blood transfusions	65 Unspecified complications	1.22 (0.72 – 2.04)	0.52
	4 Endograft occlusions	8 Endograft occlusions		
	1 Endoleak	6 Endoleaks		
	2 Acute renal failures	1 Resuscitation		
	2 Urinary tract infections	(n = 406)		
	1 Groin hematoma			
	1 Endograft infection			
	(n = 131)			
**Unfavorable Surgical Outcomes**	0 Unfavorable Surgical Outcomes	16 Reinterventions	11.73 (0.70 – 196.34)	0.01
	(n = 131)	1 Conversion to open surgery		
		(n = 409)		

The statistical analysis was made using the Fisher's Exact Test, with the assistance of RStudio version 2023.03.0. The Haldane-Anscombe correction was used for data related to unfavorable surgical outcomes.

Although our pooled analysis of complications and unfavorable outcomes included both endovascular and open surgical repairs, only a single study,^20^ in which the mean maximum diameter was ≥ 45 mm, reported results for both methods. All other studies in this review exclusively analyzed outcomes from endovascular repairs. However, this study did not specify whether the complications and unfavorable outcomes occurred in aneurysms treated with endovascular or open surgery. This methodological limitation prevented analysis of complications between these repair techniques. However, this study focused on analyzing the incidence of adverse events based on the mean diameter, aiming to determine the threshold at which outcomes would improve, regardless of the repair type.

Nevertheless, there is still no consensus on an acceptable diameter size for indicating surgical intervention. Thus, neither 45 mm nor 40 mm should be deemed definitive cut off points for deciding about SaAAA surgery. Instead, they should be used as a reference for favorable surgical outcomes, as in our results.

During the article selection process, 2 articles^22,23^ that performed radiological observation of SaAAA were found. Shang et al*.*^22^ reported a mean growth rate of 2.6 (SD, 3.2) mm/year for these aneurysms, while Bennett et al*.*^23^ reported 0.87 mm/year. This disparity in the growth rates of SaAAA suggests variation in the progression of this condition, highlighting its complex nature and management. Thus, the natural history and risk of rupture of SaAAA remain uncertain.^22^

This systematic review aimed to integrate and synthesize evidence on surgical interventions used to manage SaAAA. Our results highlight the lack of robust evidence on the surgical management of SaAAA, as evidenced by the disparity in sample sizes and significant variation in follow-up time among the included studies. This demonstrates a need for clinical trials evaluating SaAAA.

## CONCLUSIONS

Endovascular repair is the most frequent treatment option for SaAAA. Although in this review we analyzed the maximum diameter of the aneurysms – as it is the most validated parameter for the risk of rupture – the therapeutic approach should take into consideration other relevant characteristics, such as the growth rate. Additionally, individual factors, such as life expectancy and associated comorbidities, should be considered when deciding on surgical intervention.

Complications and unfavorable outcomes associated with the surgical repair of SaAAA at smaller diameters (< 40 or 45 mm) appear to be less frequent than in larger diameters (≥ 40 or 45 mm), highlighting a significant difference in unfavorable surgical outcomes when operating on mean maximum diameters < 40 mm. Furthermore, the rupture risk of these aneurysms remains uncertain. Therefore, we endorse the current notion that SaAAA should be treated electively at earlier stages to prevent the aneurysm from reaching a specific critical diameter, thereby reducing the risk of rupture and the potential for complications, as evidenced in this study. Based on such uncertainties, we will maintain this position until new studies elucidate the limits and parameters necessary to determine the right time for surgical intervention.

Due to the lack of solid evidence about how to approach SaAAAs, there is a lack of clear guidelines regarding their treatment. Additional research is needed to determine the risks, benefits, and precise indications for SaAAA interventions.
